# Blood flow restriction training on physical parameters in elite male canoe athletes

**DOI:** 10.1097/MD.0000000000035252

**Published:** 2023-10-13

**Authors:** Burcin Ugur Tosun, Ender Angin, Berkiye Kirmizigil, Mustafa Yolcu

**Affiliations:** a Department of Physical Therapy and Rehabilitation, Eastern Mediterranean University, Famagusta, Turkey; b Adana City Hospital Sports Medicine Polyclinic, Adana, Turkey.

**Keywords:** BFR, canoeing, ergometer, isokinetic strength, muscle thickness

## Abstract

**Background:**

To investigate the effect blood flow restriction (BFR) exercises on muscle size, strength and athletic performance in elite canoe athletes aged 18 to 25 years.

**Methods:**

This was a randomized controlled trial. The participants were divided into 2 groups: the intervention group (INT-gr) (n = 17, age: 18.59 ± 0.71 years) and the control group (CONT-gr) (n = 16, age: 18.81 ± 1.11 years). Anthropometric measurements, muscle size measured by ultrasound (US), strength measurements with an isokinetic dynamometer, and ergometer performance with an indoor ergometer were conducted before and after the exercise program. Knee flexion and extension and leg press one-repetition maximum (1 RM) tests were performed to determine the participants’ training program. The INT-gr performed 1 RM 30% resistance training + BFR for 8 weeks, while the CONT-gr performed 1 RM 30% resistance training (RT) without BFR with their routine training program. US was used to measure the cross sectional area (CSA) and thickness of the quadriceps femoris (QF) and Hamstring (H) muscles in the pre-post design, and the isokinetic dynamometer was used to measure the strength of bilateral 60˚/s and 300˚/s peak torque (PT) values of the QF and H. Sports performance was tested on an indoor ergometer at distances of 200, 500, and 1000 m.

**Results:**

The changes in bilateral rectus femoris (RF) CSA and VL thickness measurements in the INT-gr were significant (*P* < .05). Ergometer performance measurements showed a significant improvement over CONT-gr at all distances (*P* < .05). In terms of strength scores measured by the isokinetic dynamometer, the right QF and H 300˚/s and the left QF 60˚/s PT values were significantly in favor of INT-gr.

**Conclusion:**

BFR exercises are effective to increase strength, muscle size, and ergometer performance in elite canoe athletes.

## 1. Introduction

Canoeing has gained popularity in Turkey since the 1990s and has been an Olympic sport since 1936.^[1,2]^ As a sport characterized by the requirement for high levels of coordination, core stability, and postural control, it is imperative to acknowledge the significant values attributed to upper-extremity muscle strength and endurance, alongside the undeniable significance of lower-extremity strength and endurance. There are 3 race distances in canoeing: 200 meters (m), 500 m, and 1000 m, each of which requires different metabolic systems to be effective, including both aerobic and anaerobic capacities.^[3]^

Sporting performance refers to the total effort made to perform an athletic task.^[4]^ However, improving performance in elite athletes has become increasingly challenging due to the diverse and complex factors that influence the outcome, such as physical fitness, motor characteristics, genetic predisposition, and psychological characteristics.^[5]^ To achieve optimal performance, elite athletes must increase their physical fitness.^[4]^ Strength training is a crucial component for improving muscular fitness, which is one of the physical fitness parameters required for canoeing. The American College of Sports Medicine recommends resistance exercise with loads equal to or >65% of the one-repetition maximum (1 RM) to induce muscle hypertrophy.^[6]^ Increases in muscular strength and mass positively contribute to endurance and performance.^[7]^ Traditional weightlifting is considered the foremost approach for enhancing strength and muscle size in athletes.^[8]^ Different strategies, such as high-intensity, low-volume workout or sustained training protocols have been proven to be effective in enhancing athletes’ strength and overall capabilities.^[6]^ However, blood flow restriction (BFR) training has been gaining popularity because of its ability to produce a greater level of muscular strength and physiological adaptation at a lower intensity strength training.^[9]^

BFR training involves the performance of an exercise while external pressure restricts blood flow by wearing a pneumatic tourniquet on the proximal part of the target muscle.^[10]^ External pressure applied during BFR training restricts venous return but allows arterial blood flow. This creates an anaerobic environment during venous occlusion periods, stimulating muscle hypertrophy and causing localized, cellular, and hormonal changes. Hypoxic environments, particularly those created during exercises, increase muscle thickness and strength.^[11]^

Low-intensity resistance (LR) exercises combined with BFR training can result in effects similar to those of high-intensity resistance (HR) exercises.^[7,12]^ Consequently, BFR exercises have become a popular training method for increasing athletic capacity. This study aimed to investigate the impact of increased lower extremity strength on performance in sports such as canoeing, in which the influence of upper extremity strength and endurance is well-established. There exist no investigations regarding the effects of BFR applied to the lower extremities on sports performance and muscle thickness in elite canoe athletes. Therefore, this study aimed to determine the effects of BFR on the physical fitness parameters and ergometer performance of elite male canoe athletes and compare them with those of a control group.

## 2. Materials and methods

### 2.1. Methods

This randomized controlled study included 33 elite canoe athletes who received written and verbal information regarding the trial. The Eastern Mediterranean University Scientific Research and Health Ethics Committee approved the study on December 31, 2021 (ETK00-2022-0023) and the study was registered with clinicaltrial.gov (Identifier: NCT05225129). The study followed the principles outlined in the Declaration of Helsinki. The power and sample size of the study were evaluated using the G *Power 3.1.9.7 software.^[13]^ Based on the findings of a previous study by Luebbers et al, which reported an effect size of d = 1.205 for muscle size and strength, a sample size of 32 individuals was deemed necessary to achieve 95% power.^[14]^

**Figure 1.**
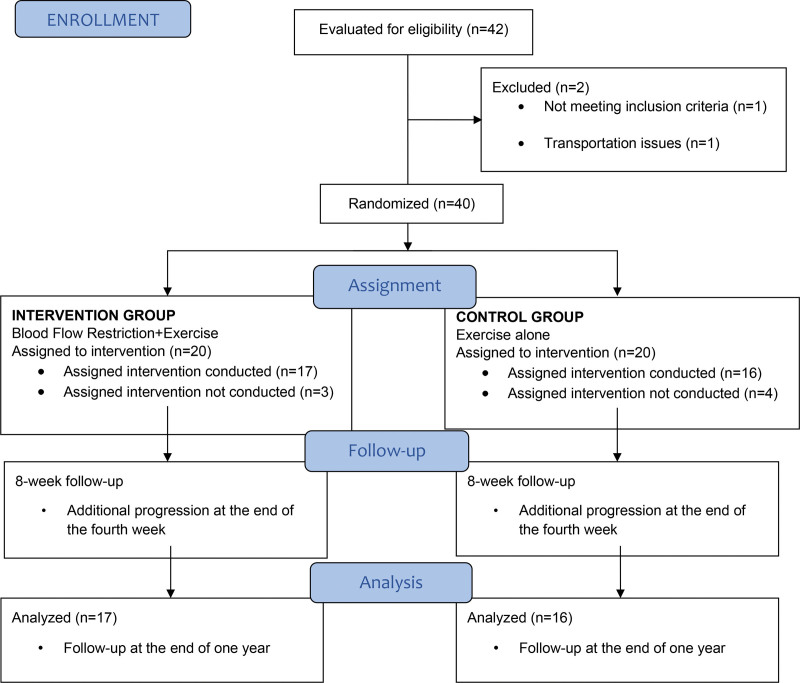
The flow diagram of the study.

Forty-two elite canoe athletes volunteered to participate in the study. The athletes were randomly divided into 2 groups: an intervention group and a control group. The intervention and control groups were composed of 40 male canoe athletes, randomly assigned using the Random Allocation Software, version 1.0. The study recruited national team athletes between 18 and 25 years of age, who were licensed canoe athletes for at least 3 years, had no history of orthopedic injury in the last 6 months, and had been performing resistance exercises for at least 5 years. Individuals with hypertension, cardiovascular disease, neurological disease, atherosclerosis, deep vein thrombosis, peripheral vascular disease, acute infection, or a history of cancer were excluded. After excluding 5 participants who could not attend regularly, one due to a sports injury, and one due to contact with COVID-19 patients, the study was completed with 33 participants. Of the 33 athletes, 17 were assigned to the intervention group (INT-gr), and the remaining 16 to the control group (CONT-gr). The athletes in both groups were assessed before and after the 8-week training program, which was conducted at the Turkish Olympic Preparation Centre in Adana Canoe-Rowing Facilities. The flow diagram of the study is shown in Figure 1.

### 2.2. Assessments

The assessments were conducted twice: before and after the training. Demographic data collection, performance tests, and muscle strength evaluations were performed by the same sports physiotherapist. The isokinetic tests were administered by a blinded physiotherapist, and the ultrasound (US) measurements were carried out by a blinded sports physician.

1. Demographic data form: The participants’ age, height, weight, years of sports experience, weekly training frequency, and arm span were recorded.

#### Physical fitness parameters

#### Sports-specific performance tests.

The rowing times for 200, 500, and 1000 m were measured using a stopwatch on a rowing ergometer and recorded in seconds (s).^[3]^ The tests for water sprint and indoor rowing showed a moderate reliability as indicated by an intraclass correlation coefficient (ICC) value of 0.69.

#### Ergometer test protocol.

The time taken to complete a specific distance was measured. Testing was conducted on a canoe ergometer designed for paddling (Dansprint PRO; Dansprint ApS, Hvidovre, Denmark). Prior to the test, each participant self-selected a 5-minute (min) warm-up session involving light cardio exercises and dynamic stretching. The paddling session commenced at the participants’ discretion in terms of rhythm, allowing them to make adjustments as desired. During this segment, the participants had control over the intensity and managed it visually. For the cool-down period, the participants were given the option to use the treadmill or the stationary bike for a duration of 10 min.

#### Muscle thickness measurements.

**Figure 2.**
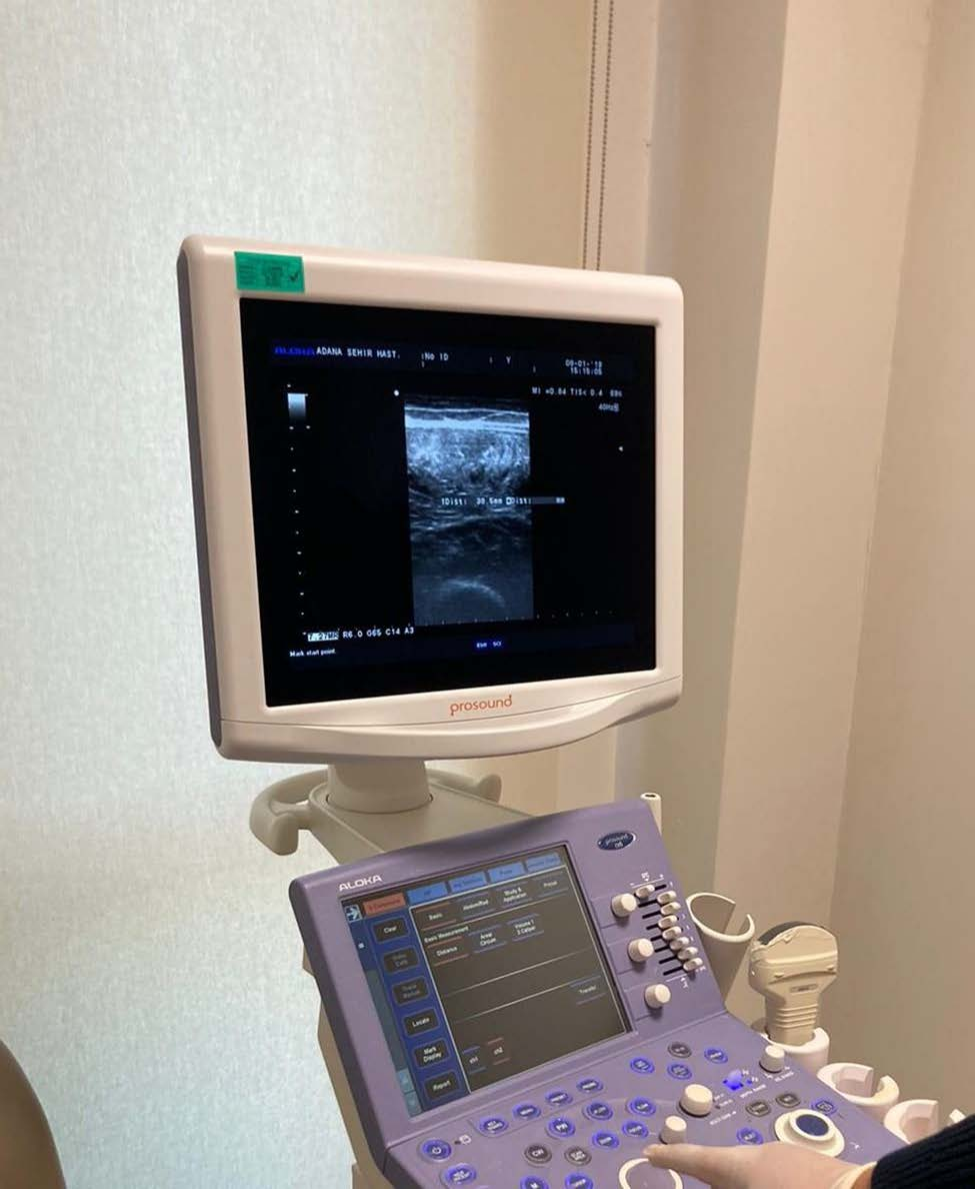
Ultrasonographic measurement of the Rectus Femoris muscle thickness.

Bilateral muscle thickness measurements were obtained for the rectus femoris (RF), vastus medialis oblique (VMO), and vastus lateralis (VL) muscles of the quadriceps femoris (QF) using the B-mode US and a 5 MHz scanning head (PROSOUND Alpha 6, Aloka, Tokyo, Japan). For optimal acoustic contact, a water-soluble transmission gel was applied to the scanning head. The scanner was placed perpendicular to the tissue interface at the predetermined marked locations. Muscle thickness was directly measured from the US display using electronic calipers, representing the distance from the interface between the adipose tissue and the muscle to the interface between muscle and bone (Fig. 2).

#### Measurement of RF.

The measurement was taken at the midpoint of the line drawn between the superior iliac anterior spine and the upper edge of the patella (ICC: 0.88).

#### Measurement of VMO.

The measurement was taken 4 centimeters (cm) above the upper edge of the patella, at a point 5 cm medial to the patella (ICC: 0.86).

#### Measurement of VL.

To measure the thickness of the VL, the midpoint of the line drawn between the superior iliac anterior spine and the upper edge of the patella was identified. Starting from this point, a measurement was taken laterally, extending by 10% in both directions (ICC: 0.83).

Each muscle group underwent 3 measurements, and the average values were documented in cm.

Moreover, the cross-sectional area (CSA) of the Hamstring (H) and RF muscles were assessed by tracing the muscle borders on 3 occasions. The resulting mean values were recorded in square centimeters (cm²).

#### Measurement of the CSA of the H.

The gluteal line was considered the proximal musculotendinous junction and was shifted distally. To determine the distal muscle-tendon connection, the probe was directed perpendicular to the long axis of the muscle, and the smallest muscle cross section was observed. This location served as the distal boundary. Measurements were obtained from the midpoint of the selected areas (ICC > 90).

#### Measurement of the CSA of the RF

A high-frequency linear US probe was placed perpendicular to the long axis of the RF muscle to achieve an optimal imaging quality. The probe was moved along the length of the muscle to identify the widest portion of the belly. Once the widest portion was identified, the boundaries of the RF muscle were manually traced using the US software. The software calculated the CSA of the RF muscle based on the traced boundaries (ICC > 80).^[10]^

#### Isokinetic test measurements

To objectively evaluate the strength of the QF and H muscles, an isokinetic test system was used (95% Confidence Interval). The peak torque (PT) ratios were measured bilaterally using a Biodex System 4XV device (USA 11967-0702) at 2 different angular velocities of 60˚/s and 300˚/s. Each athlete was instructed to perform 5 repetitions for the 60˚/s measurement and 15 repetitions for the 300˚/s measurement. Prior to the measurements, 3 repetitive trials were conducted at each angular velocity, and after a 30 seconds break, the tests were carried out. A 1-min rest was given between the 2 tests.^[15]^

### 2.3. Training protocol

### BFR training

The experimental group engaged in BFR training using KAATSU C3 equipment (KAATSUMASTER, Sato Sports Plaza Inc., Japan) on both thighs. Throughout the training sessions, the participants in the BFR group wore pneumatic cuffs positioned on the upper-thighs just below the gluteal fold. The constant mode setting was utilized during the training, via medium-sized air bands measuring 16 to 20 inches (40–51 cm) and large-sized air bands ranging from 19 to 26 inches (49–66 cm), depending on individual requirements. The pressure exerted by the KAATSU cuffs gradually increased by 10 mm Hg per session, starting at 180 mm Hg in each period and progressing to 230 mm Hg, which was then maintained in the following session. Throughout the remaining training sessions, the pressure remained constant at 230 mm Hg. The KAATSU C3 device automatically adjusted the pressure and could be attached to the athlete's body as a portable device during exercise.

### Intervention group

The subjects underwent a practice session 48 hours prior to the training. The first evaluation was performed before the 8-week training period, and the second evaluation was performed at the end of the training period. To account for potential circadian effects on performance, all measurements were performed at consistent times of day for each participant. Throughout the 8-week intervention period, both groups underwent training at identical frequencies, volumes, and intensities. To determine the maximum weight that could be lifted by the subjects, the 1 RM test was executed within the permissible range of joint motion. The participants lifted loads until their peak lifting capacity was achieved, and the 1 RM values for QF, H, and Leg Press were recorded.^[6]^ BFR exercises were combined with resistance exercises equivalent to 30% of the subjects’ 1 RM.

The sessions were conducted twice a week, with at least 48 hours between each session. The sessions commenced with a 10-min warm-up exercise on a stationary bike and continued with stretching exercises. The exercise prescription consisted of 3 sets of 10 repetitions with a 60 seconds rest between sets. The BFR device was continuously applied with pressure during the sessions, which lasted an average of 15 min. Leg Press, Leg Curl, and Quadriceps Extension machines were used for the exercises. Progression was achieved in the exercise protocol during the 4th week of the study by increasing the number of sets to 4 and the number of repetitions to 15. The sessions ended with a 10-min cool-down period of light-paced walking on the treadmill.

### Control group

The subjects underwent a practice session 48 hours before the training. Pre- and post-training evaluations were carried out to determine the QF, H, and Leg Press values using the 1 RM test. The sessions started with a 10-min warm-up exercise on a stationary bike, followed by stretching exercises. The subjects performed resistance exercises equivalent to 30% of their 1 RM twice a week for 8 weeks, with at least 48 hours between sessions. Each session lasted an average of 15 min and consisted of 3 sets of 10 repetitions with a 60 seconds rest between sets. The exercises were performed using Leg Press, Leg Curl, and Quadriceps Extension machines. Progression was achieved in the exercise protocol, with increased sets and repetitions in the 4th week of the study. The number of sets was increased to 4, and the number of repetitions was increased to 15. Finally, each session concluded with a cool-down period of 10 min of light-paced walking on the treadmill.

### Statistical analysis

Study data were analyzed using the Statistical Package for Social Sciences software (version 26.0). Age, sports age, weekly training frequency, and anthropometric measurements of both the intervention and control groups were presented as descriptive statistics. Due to the non-normal distribution of the data, the Mann–Whitney U test was employed for comparisons between the 2 groups. To compare the changes in anthropometric measurements, sport-specific performance tests, US muscle thickness measurements, and isokinetic measurements between the pretest and post-test measurements of the intervention and control groups, the ANCOVA (Analysis of Covariance) test was utilized. The level of statistical significance was set at *P* < .05 for all tests.

## 3. Results

Table 1 indicates that participants were similar in terms of age, years of sports experience, weekly training frequency, and anthropometric measurements (*P* > .05).

**Table 1:** Comparison of demographic and anthropometric characteristics of subjects.

	Group	n	*x̅*	SD	MR	Z	*P*
Age (yr)	Intervention	17	18.59	0.71	16.62	−0.259	.796
	Control	16	18.81	1.11	17.41		
Sports experience (yr)	Intervention	17	6.88	1.17	18.00	−0.655	.513
	Control	16	6.56	0.73	15.94		
Weekly number of training (d)	Intervention	17	7.24	1.64	15.24	−1.130	.258
	Control	16	7.81	1.60	18.88		
Height (cm)	Intervention	17	177.28	7.27	16.79	−0.126	.899
	Control	16	177.75	5.51	17.22		
Weight (kg)	Intervention	17	74.74	11.19	17.41	−0.252	.801
	Control	16	73.28	9.16	16.56		
Arm span (cm)	Intervention	17	178.34	7.33	16.15	−0.524	.601
	Control	16	179.69	6.35	17.91		

cm = centimeter, kg = kilogram, MR = Mann–Whitney U test statistical value, n = sample size, *x̅* = arithmetic mean, SD = standard deviation, Z = mean rank.

*P* < .05.

Table 2 shows that there was a statistically significant increase in the sport-specific performance test times of INT-gr compared to CONT-gr (*P* < .05).

**Table 2:** Inter-group comparison of subjects’ sport-specific performance tests.

	Group	Pretest		Post-test		F	*P*	Eta^2^
		*x̅*	SD	*x̅*	SD			
200 m (s)	Intervention	38.93	4.04	36.40	3.90	28.123	.000*	0.484
	Control	40.85	1.99	41.22	2.21			
500 m (s)	Intervention	110.29	8.74	104.20	8.89	30.586	.000*	0.505
	Control	114.56	4.75	117.91	5.34			
1000 m (s)	Intervention	231.29	12.10	219.59	12.02	22.927	.000*	0.433
	Control	249.44	11.89	249.59	11.32			

Eta^2^ = effect size, F = ANCOVA test statistical value, *x̅* = arithmetic mean, m = meter, s = second, SD = standard deviation.

* *P* < .05.

In Table 3, an increase in muscle thickness and CSA measurements, measured using the US, was observed in the final measurements of both groups. However, there was no significant difference between the groups (*P* > .05). The increase in bilateral RF cross-sectional area and VL thickness measurements observed in the training group was statistically higher compared to CONT-gr (*P* < .05).

**Table 3:** Inter-group comparison of US muscle thickness and cross-sectional area values of subjects.

	Group	Pretest		Post-test		F	*P*	Eta^2^
		*x̅*	SD	*x̅*	SD			
Right HCSA (cm²)	Intervention	34.86	7.10	40.43	6.85	0.968	.333	0.031
	Control	39.33	8.97	42.13	10.13			
Left HCSA (cm²)	Intervention	33.85	7.02	39.80	6.48	1.173	.287	0.038
	Control	38.01	8.68	40.63	9.26			
Right RFCSA (cm²)	Intervention	9.52	2.35	11.41	2.86	11.286	.002*	0.273
	Control	11.08	2.06	11.47	2.22			
Left RFCSA (cm²)	Intervention	8.44	2.32	10.63	2.92	8.189	.008*	0.214
	Control	11.30	2.09	11.67	2.87			
Right RFT (cm)	Intervention	1.74	0.24	1.91	0.22	1.540	.224	0.049
	Control	1.86	0.32	1.92	0.34			
Left RFT (cm)	Intervention	1.56	0.26	1.79	0.29	3.418	.074	0.102
	Control	1.80	0.37	1.84	0.33			
Right VMOT (cm)	Intervention	4.09	0.73	4.61	0.64	0.077	.784	0.003
	Control	4.15	0.82	4.60	0.73			
Left VMOT (cm)	Intervention	3.76	0.70	4.17	0.57	1.490	.232	0.047
	Control	4.20	0.61	4.32	0.65			
Right VLT (cm)	Intervention	1.92	0.28	2.36	0.43	9.852	.004*	0.247
	Control	2.15	0.24	2.15	0.32			
Left VLT (cm)	Intervention	1.79	0.30	2.25	0.46	7.451	.011*	0.199
	Control	2.01	0.27	2.04	0.28			

cm = centimeter, cm² = square centimeter, Eta^2^ = effect size, F = ANCOVA test statistical value, *x̅* = arithmetic mean, HCSA = hamstring cross-sectional area, RFCSA = rectus femoris cross-sectional area, RFT = rectus femoris thickness, SD = standard deviation, VLT = vastus lateralis thickness, VMOT = vastus medialis oblique thickness.

* *P* < .05.

Finally, as shown in Table 4, the changes between pre- and post-training right QF and H 300˚/s PT measurements and the increase in left QF measurements at 60˚/s PT were statistically significant in INT-gr compared to controls (*P* < .05).

**Table 4:** Inter-group comparison of isokinetic test results of subjects.

	Group	Pretest		Posttest		F	*P*	Eta^2^
		*x̅*	SD	*x̅*	SD			
Right QF 60˚ PT	Intervention	198.16	36.58	227.12	46.91	1.402	.246	0.045
	Control	205.30	52.75	220.11	58.80			
Left QF 60˚ PT	Intervention	191.52	27.33	224.45	42.51	6.347	.017*	0.175
	Control	210.34	45.66	216.38	40.43			
Right QF 300˚ PT	Intervention	97.29	24.32	112.63	19.40	4.570	.041*	0.132
	Control	101.29	24.07	104.10	17.33			
Left QF 300˚ PT	Intervention	93.97	18.87	109.37	18.03	0.000	.989	0.000
	Control	103.07	32.28	118.90	39.18			
Right H 60˚ PT	Intervention	111.90	46.12	132.51	30.97	3.247	.082	0.098
	Control	108.41	58.27	116.27	27.41			
Left H 60˚ PT	Intervention	110.08	42.38	125.62	27.90	0.680	.416	0.022
	Control	118.96	46.93	139.54	50.14			
Right H 300˚ PT	Intervention	63.05	27.83	88.66	23.34	4.442	.044*	0.129
	Control	66.13	22.23	76.53	15.27			
Left H 300˚ PT	Intervention	66.94	22.26	84.38	17.74	1.893	.179	0.059
	Control	65.13	16.86	76.29	17.15			

˚ = degree, Eta^2^ = effect size, F = ANCOVA test statistical value, H = Hamstring, *x̅* = arithmetic mean, PT = peak torque, QF = quadriceps femoris, SD = standard deviation.

* *P* < .05.

## 4. Discussion

In the present study, the effects of BFR training on enhancing the strength of often-overlooked lower extremity muscles in canoeing as well as the level of their contribution to ergometer performance were investigated. Whether gains would be achieved through LR exercises over a short period has a positive impact on the performance of canoe athletes was also investigated. The results of the study revealed that BFR training increased muscle thickness and strength in elite male canoe athletes compared to controls. The ergometer performance times significantly reduced as measured by sport-specific tests. Although no difference was observed in the development of the H muscles in the US CSA measurements between the groups, there was an increase in the RF muscle. The isokinetic measurement results did not show any significant differences among the right QF 60˚/s, left H 60˚/s, and left H 300˚/s PT in both groups. However, there was improvement in the left QF 60˚/s, right QF 300˚/s, and right and left H 300˚/s PT in both groups.

Canoeing is a sport that requires strength and endurance in the upper and lower extremities as well as core strength and stability, especially due to the athlete precarious posture in a narrow boat.^[16]^ In addition to the trunk muscles, upper limb muscles also play a role in propelling the boat forward.^[17]^ A previous study suggested that the evaluation of upper-extremity muscle strength and endurance alone in canoe athletes was insufficient and that the lower extremities should also be considered.^[18]^ Hamano et al investigated the relationship between body composition and cycling power output in canoe paddlers. The research indicated that the muscle mass of the lower extremities plays an important role in enhancing both lean body mass and performance in canoe sprinting. The results of the performance test were consistent with isokinetic knee extension and flexion strength in canoe paddlers.^[19]^ Both upper and lower extremity strength in canoeing are important and the resistance training (RT) is effective in athletic development. Therefore, it is valuable to investigate the effects of RT, specifically BFR exercises, on the lower extremity muscles of elite canoeists.

RT is commonly used to promote athletic development and it helps athletes meet the demands of their sport branches.^[20]^ These exercises stimulate type 2 muscle fibers in the skeletal muscles, which can increase both strength and endurance.^[21]^ One study suggested that combining BFR with LR training produced the highest strength and muscle mass increase.^[22]^ The times achieved on the ergometer for the 200 and 2000 m canoe distances reflect on water performance values. The effect of lower extremity pushing force on canoe performance is critical, as it enables the canoeist to move faster.^[23]^ Another study investigating the connection between muscle mass and performance in canoeists found that each 1 kg increase in mass improved performance by reducing race times by 1.32 seconds at 500 m and 6.80 seconds at 2000 m.^[24]^ According to another study, an increase in mass led to a decrease in the 2000 m race time, but there was no change in the race time for the 200 to 250 m distances.^[25]^ Our results agree with those reported in the literature because it was found that an increase in strength was associated with improved performance among canoeists.

In recent years, US has gained popularity for the measurement of skeletal muscle thickness and CSA.^[26]^ In 2012, an occlusion study evaluated the CSAs of athletes’ QF and H muscles using US. The experimental group showed a muscle mass increase of 4% to 12%, with QF displaying a greater increase than H. Despite equal pressure being applied to the knee joint during exercises, the development of the QF and H muscles varied, possibly due to differences in the exercise position.^[27]^ In a 3-week BFR study, MR (magnetic resonance imaging) measurements showed a 5.3% increase in the mass of the QF and H muscles. However, isometric muscle strength measurements indicate an increase only in QF values.^[28]^ No significant differences were found in the growth of the H muscles between the 2 groups, as CSA measurement by US. However, there was an increase in the RF muscle size. Moreover, there was no significant growth in the thickness measurements of the RF and VMO muscles; however, the thickness of the VL muscle was significantly greater. The RF and VL, 2 of the 4 QF muscles, are the most frequently used muscles in canoeing, and have been emphasized in the literature as necessary for good joint control.^[29]^ Overall, increasing the QF muscle size, particularly the RF and VL, can improve joint control, enhance power generation, maintain a stable bracing position, and prevent canoeing injuries.

Yasuda et al measured the effects of a hypoxic and acidic environment on muscles using electromyography. This environment can inhibit both current muscle contraction and the potential for muscle contraction, which is relevant to BFR training. As suggested in the literature, occlusion-induced hypoxic and acidic environments can promote permanent muscle hypertrophy by forming additional fast-contracting motor units.^[30]^ This phenomenon can be considered the primary reason for the gains observed during training.

In the present study, the force generated by canoe athletes was evaluated using an isokinetic dynamometer. The results showed that the increase in PT values in the BFR group was higher than that in CONT-gr. The higher force increase in INT-gr may be due to the hypoxic environment created by BFR, as noted in a previous study by Yasuda et al.^[30]^ Regarding isokinetic measurements, no significant differences were found in the present study between the groups in the right QF 60˚/s, left H 60˚/s, and left H 300˚/s PT. However, left QF 60˚/s, right QF 300˚/s, right H 300˚/s, and left H 300˚/s PT showed improvement in both groups, indicating that there was an increase in the strength of these muscles in INT-gr, but the same level of improvement did not occur in CONT-gr. The differences observed in the isokinetic measurements could be attributed to the use of BFR and participants’ physical abilities.

BFR exercises offer a viable alternative in cases where resistance exercises are not recommended. Progressive RT, which involves adjusting the external load, sets, and repetitions, is known to promote strength and muscle size development.^[31]^ However, performing HR shortly after injury or surgery can improve tissue recovery. BFR training typically involves lower loads (20%–30% of 1-RM; 15 to 30 repetitions per set) and effectively addresses weakness and muscle wasting without overwhelming the recovering tissues.^[8,32]^ The results of this study support the use of BFR as a therapeutic tool for improving physical health and well-being. Combining moderate resistance of low weights with occlusion training provides an opportunity to maintain or even increase muscle size by creating a hypoxic environment, and thereby prevents performance decline.

## 5. Limitations

The study included a relatively small sample size and only male athletes aged 18 to 25 years. The fact that the participants were elite athletes limits the sample size.

## 6. Conclusions

In conclusion, increasing lower extremity muscle size and strength yielded a positive impact on the athletic performance of canoe athletes compared with CONT-gr. BFR exercises can be used as a strengthening method, in addition to sports training. By combining BFR training with low-intensity exercises, it is possible to achieve faster muscle thickness and strength gains in the RF and VL muscles compared to normal training durations.

## Acknowledgments

The authors would like to thank Eastern Mediterranean University for supporting them.

## Author contributions

**Conceptualization:** Burcin Ugur Tosun, Ender Angin.

**Data curation:** Burcin Ugur Tosun.

**Formal analysis:** Burcin Ugur Tosun, Ender Angin, Mustafa Yolcu.

**Investigation:** Burcin Ugur Tosun, Ender Angin.

**Methodology:** Burcin Ugur Tosun, Ender Angin, Berkiye Kirmizigil, Mustafa Yolcu.

**Resources:** Mustafa Yolcu.

**Software:** Mustafa Yolcu.

**Supervision:** Berkiye Kirmizigil, Mustafa Yolcu.

**Visualization:** Ender Angin, Berkiye Kirmizigil.

**Writing – original draft:** Burcin Ugur Tosun.

**Writing – review & editing:** Berkiye Kirmizigil.
